# Multimodal machine learning-based model for differentiating nontuberculous mycobacteria from *mycobacterium tuberculosis*

**DOI:** 10.3389/fpubh.2025.1470072

**Published:** 2025-02-17

**Authors:** Hong-ling Li, Ri-zeng Zhi, Hua-sheng Liu, Mei Wang, Si-jie Yu

**Affiliations:** Department of Infectious Diseases, Zhoushan Hospital, Wenzhou Medical University, Zhoushan, Zhejiang, China

**Keywords:** nontuberculous mycobacterium, *mycobacterium tuberculosis*, deep learning, CT images, multimodal model

## Abstract

**Objective:**

To develop and evaluate the effectiveness of multimodal machine learning approach for the differentiation of NTM from MTB.

**Methods:**

The clinical data and CT images of 175 patients were retrospectively obtained. We established clinical data-based model, radiomics-based model, and multimodal (clinical plus radiomics) model gradually using 5 machine learning algorithms (Logistic, XGBoost, AdaBoost, RandomForest, and LightGBM). Optimal algorithm in each model was selected after evaluating the differentiation performance both in training and validation sets. The model performance was further verified using external new MTB and NTM patient data. Performance was also compared with the existing approaches and model.

**Results:**

The clinical data-based model contained age, gender, and IL-6, and the RandomForest algorithm achieved the optimal learning model. Two key radiomics features of CT images were identified and then used to establish the radiomics model, finding that model from Logistic algorithm was the optimal. The multimodal model contained age, IL-6, and the 2 radiomics features, and the optimal model was from LightGBM algorithm. The optimal multimodal model had the highest AUC value, accuracy, sensitivity, and negative predictive value compared with the optimal clinical or radiomics models, and its’ favorable performance was also verified in the external test dataset (accuracy = 0.745, sensitivity = 0.900). Additionally, the performance of multimodal model was better than that of the radiologist, NGS detection, and existing machine learning model, with an increased accuracy of 26, 4, and 6%, respectively.

**Conclusion:**

This is the first study to establish multimodal model to distinguish NTM from MTB and it performs well in differentiating them, which has the potential to aid clinical decision-making for experienced radiologists.

## 1 Introduction

Tuberculosis is a chronic infectious disease caused by *mycobacterium tuberculosis* (MTB). MTB can invade all organs of the body, especially lung. Tuberculosis caused by MTB is one of the most serious public health problems in the world (1). The tuberculosis burden in China is only lower than in India and Indonesia, ranking third (2). With the increasing number of patients with human immunodeficiency virus (HIV) year by year and the massive use of immunosuppressants, the incidence of opportunistic infections and disease burden caused by non-tuberculous mycobacteria (NTM) are rising globally (3, 4). NTM is a major cause of morbidity and mortality in progressive lung diseases. However, the clinical manifestations of MTB and NTM are similar in symptoms such as low fever, cough, and reduced body weight (5), making it difficult to distinguish them. Therefore, choosing a fast, accurate, and clinically applicable method for distinguishing NTM and MTB is of great significance.

The bacteria culture is the “gold” reference standard and takes 2 to 6 weeks to produce diagnostic results. However, due to the different treatment plans between the two types of diseases, NTM patients will miss the best treatment opportunity due to the long time consumption, leading to disease progression (6). In addition, bacterial culture shows low sensitivity. Other methods (7) including GeneXpert MTB/RIF Ultra, chest X-ray, and tuberculosis loop-mediated isothermal amplification fail to distinguish between NTM and MTB. Recently, next-generation sequencing (NGS) based technology has been successfully applied for the routine characterization of tuberculosis (8, 9). Therefore, it is extremely necessary to combine multiple methods for the differentiation between NTM and MTB.

Although chest computed tomography (CT) examination also faced a similar predicament of difficulty in identification, there are still subtle differences in imaging performance between the two diseases (10). This indicates that CT signs may play an important role in disease differentiation and it is necessary to mine more useful information behind CT images. In recent years, radiomics has shown great potential in the diagnosis and differential diagnosis of lung diseases through high-throughput extraction and mining of data features (11, 12), which may provide a feasible method for distinguishing NTM from MTB. Radiomics is a non-invasive and objective image analysis tool, that uses computer algorithms to mine deep information in images such as CT, Magnetic Resonance Imaging (MRI), and positron emission tomography (PET), thereby reflecting the heterogeneity of the lesion area. However, the discriminating model based on these imaging pictures showed different differentiation performances. For example, the previous study quantifies bronchiectasis regions in CT images and explored a machine learning approach, finding that the model achieved an area under the curve (AUC) of 0.84 and an accuracy of 0.85 (13). However, the other research reported an accuracy of 0.74 (14). It followed that the performance difference between different machine learning models was the key point when applying these models for distinguishing lung diseases.

Machine learning (ML) is a technique, which can automatically extract useful models from large-scale heterogeneous datasets based on complex algorithms. These models can then be utilized for outcome prediction (15). ML has enhanced the integration of computer science and statistics with medical problems, and it is now extensively employed in disease diagnosis, cancer treatment, and other medical research areas (16–18). In tuberculosis research, ML is also widely applied for the diagnosis, treatment and differential diagnosis of tuberculosis. Yao et al. (19) combined plasma proteins with ML to establish seven models for the diagnosis of active tuberculosis. Among them, the support vector machine (SVM) model demonstrated the best performance, achieving an AUC exceeding 0.89. In addition, ML in conjunction with histological information can diagnose latent tuberculosis (20). Research has shown that the ML fusion model based on longitudinal CT scan image histology performs well in predicting the poor prognosis of TB treatment, with internally validated AUC and externally validated AUC of 0.767–0.802 and 0.831–0.857, respectively, enabling early preventive measures against unfavorable prognoses (21). Although some researchers have also built models to differentiate between NTM and MTB by combining urinary metabolomics or CT imaging information with ML (22, 23), these models are limited in their clinical interpretability due to the only utilization of single laboratory or CT imaging parameter. Currently, limited researches performed machine learning to distinguish NTM from MTB, especially from aspect of multidimensions (clinical characteristics, laboratory test, CT/MRI images, etc.).

In this study, we conducted five machine learning algorithms and established an optimal multimodal model containing clinical, laboratory test, and radiomics data of CT images for distinguishing NTM from MTB. We compared its differentiation performance with single clinical or radiomics based models. We also verified its differentiation performance in the external new dataset, and then compared its’ differentiation ability model with the existing approaches and machine learning model. The contributions of our study were as follows: (1) at present, few study was performed for distinguishing them using machine learning models. Our study aimed to establish a machine learning model for differentiating NTM from MTB, which can promote the application of new technologies, thereby advancing research progress in this fields. (2) Currently, most of studies were based on the one dimension, such as only CT or X-ray images. This is the first study to consider multidimensions (clinical characteristics, laboratory test, CT images) and use multimodal model to distinguish them. Our research provides a new perspective and strategy for differentiating NTM from MTB, which is of great significance for doctors to choose appropriate treatment plans.

## 2 Methods

### 2.1 Data source

This study retrospectively enrolled patients with pulmonary infection who were admitted to our hospital between April 2020 and December 2023. All the patients underwent a CT examination.

The diagnostic criteria were presented as follows. NTM was diagnosed according to the Euro-American 2020 edition (24). MTB was diagnosed according to the rapid tuberculosis diagnostic criteria with 2023 World Health Organization edition (25). To differentiate the NTM and MTB, we conducted the T-SPOT test using a testing kit produced by DEAOU (Guangzhou) according to the kit instructions. Next-generation sequencing (NGS) of alveolar lavage fluid and bacteria culture were also performed to differentiate the NTM and MTB. The collection of alveolar lavage fluid and next-generation sequencing information including sample processing and DNA extraction, and library generation and sequencing can be found in the previous study (26). Integrating the results of multiple tests, the NTM and MTB patients were classified.

**Figure 1:**
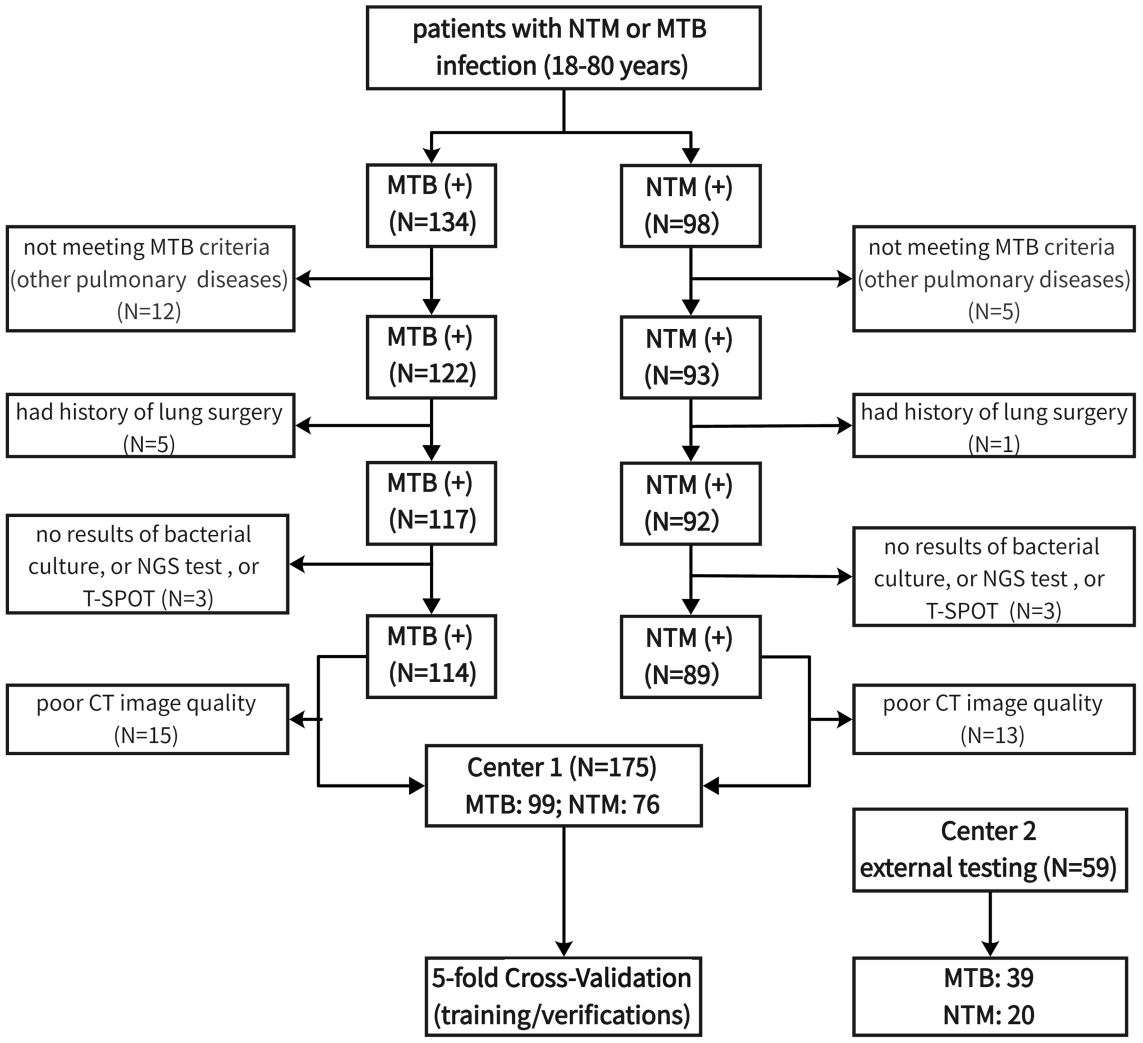
The process of patient screening and enrolling.

The inclusion criteria included (1) age ranged from 18 to 80 years; (2) diagnosed with NTM or MTB infection; (3) had bacterial culture results; (4) had NGS test results; (5) had T-SPOT test results; (6) with at least 2 set of lung CT images available. We excluded these patients with lung cancer, fungal infection, pneumoconiosis, and mixed infections of TB and NTM. The process of patient screening and enrolling was presented in Figure 1. After screening, 99 MTB patients and 76 NTM patients were enrolled in the final analyses.

It should be noted that there was a data imbalance regarding the MTB and NTM patient number, which may cause the potential for producing overfitting problems. Due to the requirements of periodical for follow-up after diagnosis, at least 2 images of NTM/MTB patients were taken. Considering the slow rate of radiological abnormal changes in NTM, longer follow-up time was required. Hence, more CT images of one NTM patient were collected than MTB. Therefore, although there was unbalance in the proportion of patients, CT images were as balanced as possible.

### 2.2 Machine learning (ML) approach

**Figure 2:**
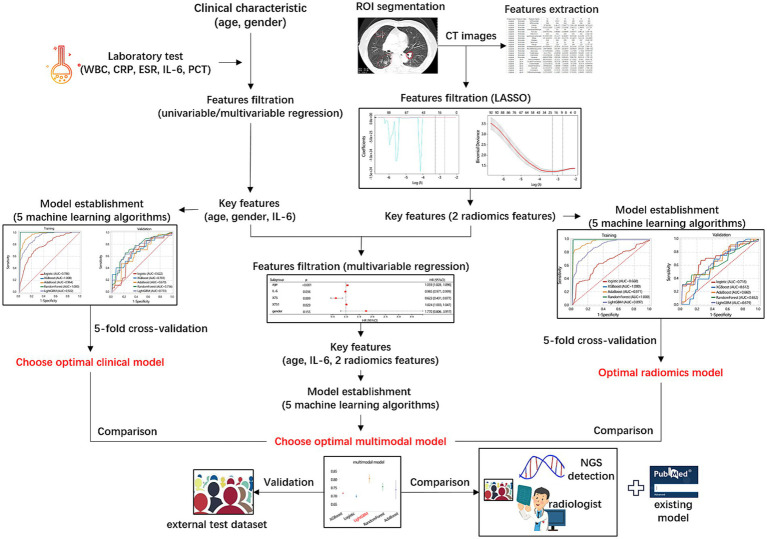
Overall workflow of the models’ development, validation, and comparison.

This study aimed to construct 3 models (clinical model, radiomics model, multimodal model) for differentiating NTM from MTB using 5 machine learning algorithms (Logistic, XGBoost, AdaBoost, RandomForest, and LightGBM), and to explore which algorithms was more suitable. The purpose of classified multi-model method was to select the best model, rather than directly modeling to get the final model. In this study, we used the training/verification mechanism of 5-fold cross-validation to summarize the performance of each model in many trainings, focusing on the overall performance of each model. The overall workflow of the models’ development, validation, and comparison was presented in Figure 2.

Logistic regression models are more traditional single-model classification algorithms that aim to identify the connection between features and the likelihood of a specific (binary) outcome. It is widely employed by medical professionals for its ability to calculate odds ratio (27).

Models such as RandomForest, XGBoost, LightGBM, and AdaBoost are ensemble models based on decision trees. These ensemble models can get a strong model through taking the strengths of all single models. This strong classifier achieves a relative best performance.

RandomForest algorithm is the embodiment of group intelligence, which creates different training sets by randomly sampling rows (bagging) and columns (feature bagging) from the dataset. The decision tree of RandomForest algorithm is grown by bootstrap. RandomForest reduces training variance and improves model generalization and integration. And it can be utilized without parameter tuning, offering variable importance information for classification and high predictive accuracy (28).

Conversely, XGBoost, LightGBM, and AdaBoost are based on the idea of gradient boosting. XGBoost performs a second-order Taylor expansion of the loss function and employs various techniques to minimize overfitting. It utilizes the structure score (gain) of the tree to determine split points, improving tree quality, and implements parallel and distributed computation for increased efficiency. For detailed algorithm information, consult the literature (29).

The LightGBM algorithm employs a histogram approximation algorithm to generate a histogram that discretises continuous features. It splits trees at leaf nodes with the largest lift. And using the GOSS technique, the samples with larger gradient are preferred, so the optimal split can be found faster and the training efficiency is improved (30, 31).

AdaBoost is a relatively new nonlinear ML algorithm that builds a tree by adjusting sample weights and combines multiple trees to become a strong classifier. It does not require feature screening, can perform automatic feature selection, and has a low risk of overfitting. For specific steps, refer to the relevant literature (32).

### 2.3 Key clinical features identification

We collected the clinical data of patients including the age (years), gender, white blood cell (WBC, 10^9^/L), erythrocyte sedimentation rate (ESR, mm/h), C-reactive protein (CRP, mg/L), Interleukin-6 (IL-6, pg./mL), and procalcitonin (PCT, ng/mL). The difference in these variables between NTM and MTB groups was first compared to identify the differential variables. These differential variables were then enrolled in Logistic regression analysis to determine the independent factor associated with the disease types. Then Receiver Operating Characteristic (ROC) analysis and area under curve (AUC) were used to assess the discriminating performance of these independent factors. Decision Curve Analysis (DCA) was used to evaluate their obtained clinical net benefit for discriminating disease.

### 2.4 Differentiation model construction based on the key clinical features

Based on the independent clinical factors, a clinical features-based model for discriminating NTM from MTB was first constructed and validated using 5-fold cross-validation. The cross-validation can partially resolve the overfitting problem. In addition, we added L2 penalty to control the complexity of the model to prevent it from being too complicated. We also applied early stop-ping, learning rate adjustments, and drop-out to prevent overfitting. The detailed parameters of the Logistic algorithm were as follows: *C* = 1.0; max-iter = 100; penalty = l2; tol = 0.0001. The parameters of the XGBoost algorithm were as follows: learning-rate = None; max-depth = None; min child-weight = None; reg-lambda = None. The parameters of the AdaBoost algorithm were as follows: learning-rate = 1.0; n-estimators = 50. The parameters of the RandomForest algorithm were as follows: criterion = gini; max-depth = None; min impurity-decrease = 0.0; n-estimators = 20. The parameters of the LightGBM algorithm were as follows: boosting type = gbdt; learning rate = 0.1; max depth = −1; n-estimators = 100; num-leaves = 31. The discriminating performance of models from 5 algorithms was evaluated by ROC and DCA analyses.

### 2.5 CT images and radiomics features extraction

The CT images of all patients were also obtained. CT scans were performed using 64-slice CT scanners with the following parameters: tube voltage 120 kV; automatic tube current modulation 300 mA; detector collimation 64 × 0.625 mm; thread pitch 0.993; section thickness 2 mm; section interval 2 mm. The scanning area ranged from the apex pulmonic to the bottom of the lung.

All CT images were assessed by two radiologists with 5 years of diagnostic experience in CT, respectively. They were blinded to the histopathological and clinical data of patients. The 2 radiologists manually segmented the region of interest (ROI) of CT images by using 3D slicer software, and the intraclass correlation coefficient (ICC) between the 2 radiologists indicated the consistency of extracted features. ICC > 8 suggested a good consistency between observers.

For feature extraction, all images and ROIs were batched into 3D slicer software. The extracted radiomics features included 162 first-order features, 216 gray-level co-occurrence matrix (GLCM) features, 126 gray-level dependence matrix (GLDM) features, 144 gray-level run-length matrix (GLRLM) features, 144 gray-level size zone matrix (GLSZM) features, and 45 neighboring gray-tone difference matrix (NGTDM) features.

### 2.6 Differentiation model construction based on radiomics features of CT images

A total of 837 radiomics features were extracted. We then used the Least absolute shrinkage and selection operator (LASSO) analysis to remove the redundant features. By introducing a penalty coefficient (*λ*), the coefficients of most features will be compressed to 0. The retained features in the final optimal LASSO model were selected for further analysis. LASSO analysis is a regularization method for regression analysis. By introducing L1 regularization into the regression model, the coefficients of some features are reduced to zero, thus realizing variable selection. LAASO can reduce the complexity of the model, and improve the prediction performance of the model. Therefore, application of LASSO analysis can partially resolve the overfitting problem. Then, the differences of the retained features after LASSO analysis between NTM and MTB groups were compared. The differential features between the 2 groups were enrolled in the logistic regression analysis to explore the independent factors. The clinical value of independent factors in disease discrimination was assessed by ROC and DCA analysis.

Based on the independent features, a radiomics features-based differentiation model was constructed in a training set using 5 machine learning algorithms including Logistic, XGBoost, AdaBoost, RandomForest, and LightGBM. Their performance was also verified in the validation set by ROC and DCA analyses. Detailed information for the model construction and validation can be found in the Methods 2.3 section.

### 2.7 Multimodal differentiation model construction

The key clinical and radiomics features, that are independently related to the disease types, have been identified in the above analyses. Then these various indicators were enrolled in logistic regression analysis to further determine the independent variables. Based on the key clinical and radiomics features, a multimodal differentiation model was constructed using 5 machine learning algorithms. Their performance was also verified in the validation set by ROC and DCA analyses. Detailed information for the model construction and validation can be found in the Methods 2.3 section. Especially, the importance ranking of features within the multimodal model was explored using 3 optimal algorithms.

### 2.8 Differentiation performance comparison and verification

Then, the differentiation performance of the 3 models (clinical-based, radiomics-based, multimodal-based) was compared from aspects of AUC, cutoff, accuracy, sensitivity, specificity, positive predictive value (PPV), negative predictive value (NPV), and Kappa values. Although we have collected data for 4 years to establish and evaluate our models, there was limit to accurately assessing their performance due to the lack of test data, especially for NTM. Therefore, it was necessary to obtain a new external dataset from public data or data from other institutions. Unfortunately, we failed to obtain the test data from other institutions due to some reasons. Currently, no publica data on NTM was provided.

Therefore, another external dataset containing NTM and MTB cases who met the diagnostic criteria were retrospectively found. From January 2024 to December 2024, totally 59 patients (20 NTM and 39 MTB patients) were collected. These data differed from the patient data used when constructing the learning model, and were the newly generated data after performing learning model. Only one image was collected per patient in the new external dataset. We also validated the performance of our models for differentiating NTM and MTB using the new external test dataset. The differentiation performances of 3 models were also compared in the new external test dataset.

In addition, we compared the differentiation performance of multimodal model in the new external test dataset with other measures including NGS detection method, radiologist assessment with 5-year experiences, and existing machine learning model. In terms of radiologist assessment, one radiologist identified the pathogen that infected the patients in the testing dataset by only scanning CT images without any other reminding. The radiologist was informed that the patients were only infected by one type of pathogen, either NTM or MTB. The existing machine learning model was searched from the published articles. Currently, few studies reported the machine learning model for the differentiation of NTM from MTB. Finally, a deep learning model based on CT images by Wang et al. was selected for the performance comparison (33). For the differentiation performance comparison among different methods, AUC, accuracy, sensitivity, specificity, MTB-precision, and NTM-precision were set as the indicators.

### 2.9 Statistical analysis

The continuous variables were presented as median and quartiles as they did not confirm to the normal distribution, and their differences between the 2 groups were compared with the Mann–Whitney U test. The categorical variables were expressed as frequency and percent, and their distribution difference between the 2 groups was analyzed by *χ*^2^ test. Univariable and multivariable logistic regression analyses were used to explore the association between variables and disease types. ROC and DCA were used to assess the discriminating performance. *p* < 0.05 was considered statistical significance.

## 3 Results

### 3.1 Characteristics of patients

In this study, we enrolled 175 patients infected by NMT or MTB to our analysis the median age was 63 [51, 70] years old, with average age of 58 years old. The median white blood cell was 5.6 [4.7, 7.2] (10^9^/L). The median CRP was 4.4 [1.3, 21.0] (mg/L). The median ESR was 32.0 [12.5, 59.5] (mg/L). The median IL-6 was 26.65 [4.38, 56.22] (pg/mL). Male patients accounted for 54% of all patients. Of the 76 patients diagnosed with NTM, 35 patients (46.1%), 23 patients (30.2%), 15 patients (19.7%), and 3 patients (4.0%) were infected by *M. avium*, *M. intracellulare*, *M. abscessus*, and *M. kansasii* strains of NTM, respectively.

The baseline characteristics of MTB (*N* = 99) and NTM (*N* = 76) patients are presented in Table 1. It followed that age (*p* < 0.001), gender (*p* = 0.002), and IL-6 level (*p* < 0.001) showed significant differences between MTB and NTM groups. NTM group had higher median age (66 vs. 57), but lower WBC (5.4 vs. 5.9), CRP (4.4 vs. 5.0), ESR (27 vs. 32), and IL-6 (15.6 vs. 39.5) levels than MTB group.

**Table 1:** The baseline characteristics of patients grouped by infection types.

		MTB (*n* = 99)	NTM (*n* = 76)	*p*
Age (years)		57 [38, 69]	66 [59, 72]	<0.001
WBC (10^9^/L)		5.9 [4.8, 7.3]	5.4 [4.5, 6.8]	0.109
CRP (mg/L)		5.0 [1.7, 24.7]	4.4 [1.3, 15.9]	0.323
ESR (mm/h)		32 [15, 62]	27 [12, 57]	0.779
IL-6 (pg/mL)		39.5 [16.0, 60.8]	15.6 [2.5, 45.9]	<0.001
Gender	Male	64 (64.646)	31 (40.789)	0.002
	Female	35 (35.354)	45 (59.211)	
PCT (ng/mL)	<0.01	7 (7.071)	10 (13.158)	0.178
	≥0.01	92 (92.929)	66 (86.842)	

WBC, white blood cell; ESR, erythrocyte sedimentation rate; CRP, C-reactive protein; PCT, procalcitonin.

### 3.2 Clinical feature-based differentiation model

**Figure 3:**
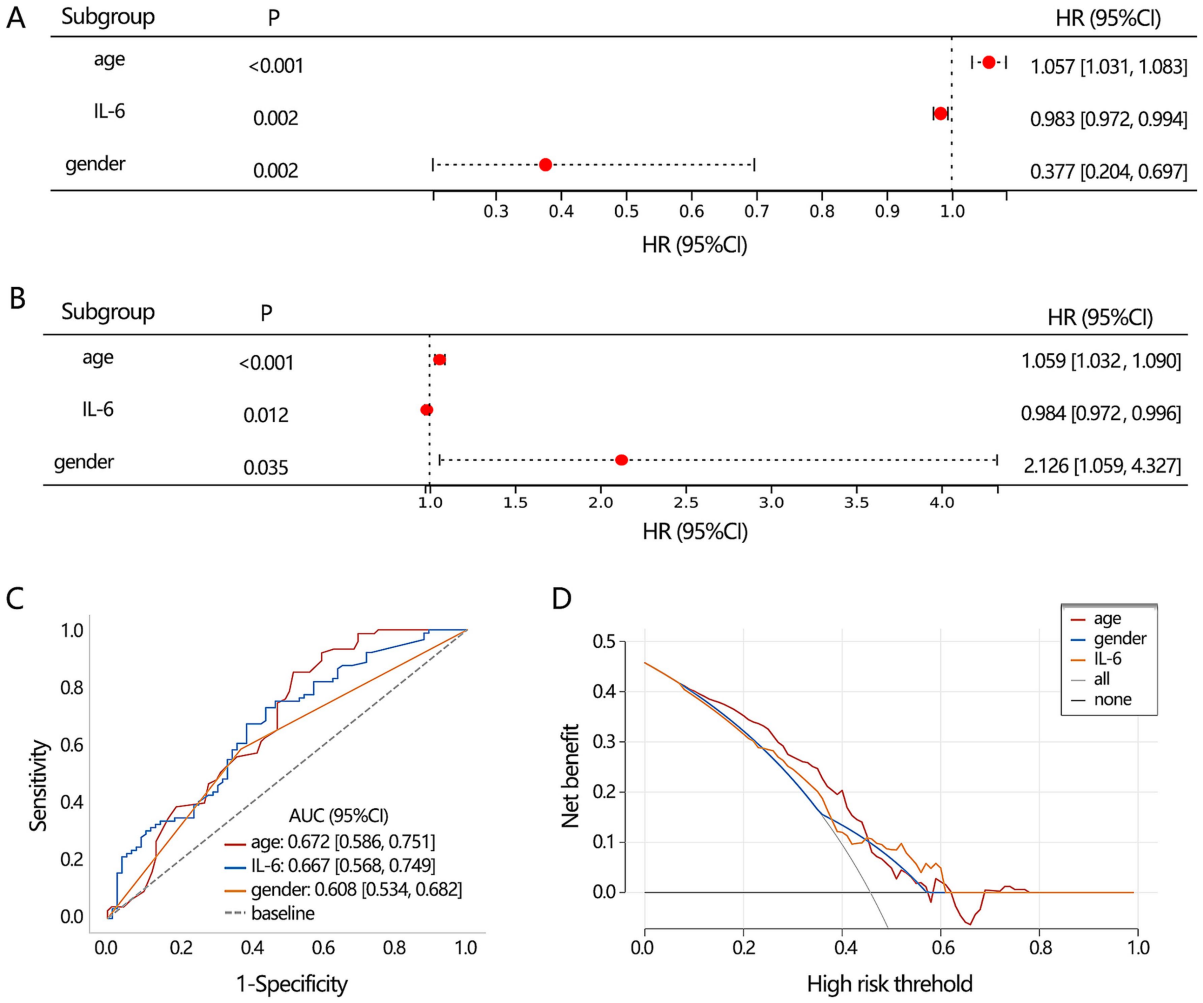
The differentiation value assessment on the significant clinical indicators. **(A)** Univariable and **(B)** multivariable logistic regression analyses. **(C)** ROC analysis was conducted to assess the discriminating performance of TB and NTM. **(D)** DCA analysis was used to assess the clinical net benefit.

Based on 3 significant baseline characteristics, we next explored their association with the disease types. The univariable logistic regression presented their significant association (Figure 3A, all *p* < 0.05), and the multivariable regression analysis further displayed their independent association (Figure 3B, all *p* < 0.05). Their differential performance of TB and NTM was then assessed by ROC analysis (Figure 3C), finding that age had the highest AUC value (0.672), followed by IL-6 (0.667). DCA analysis showed that the 3 clinical features obtained similar clinical net benefits for discriminating TB and NTM (Figure 3D).

**Figure 4:**
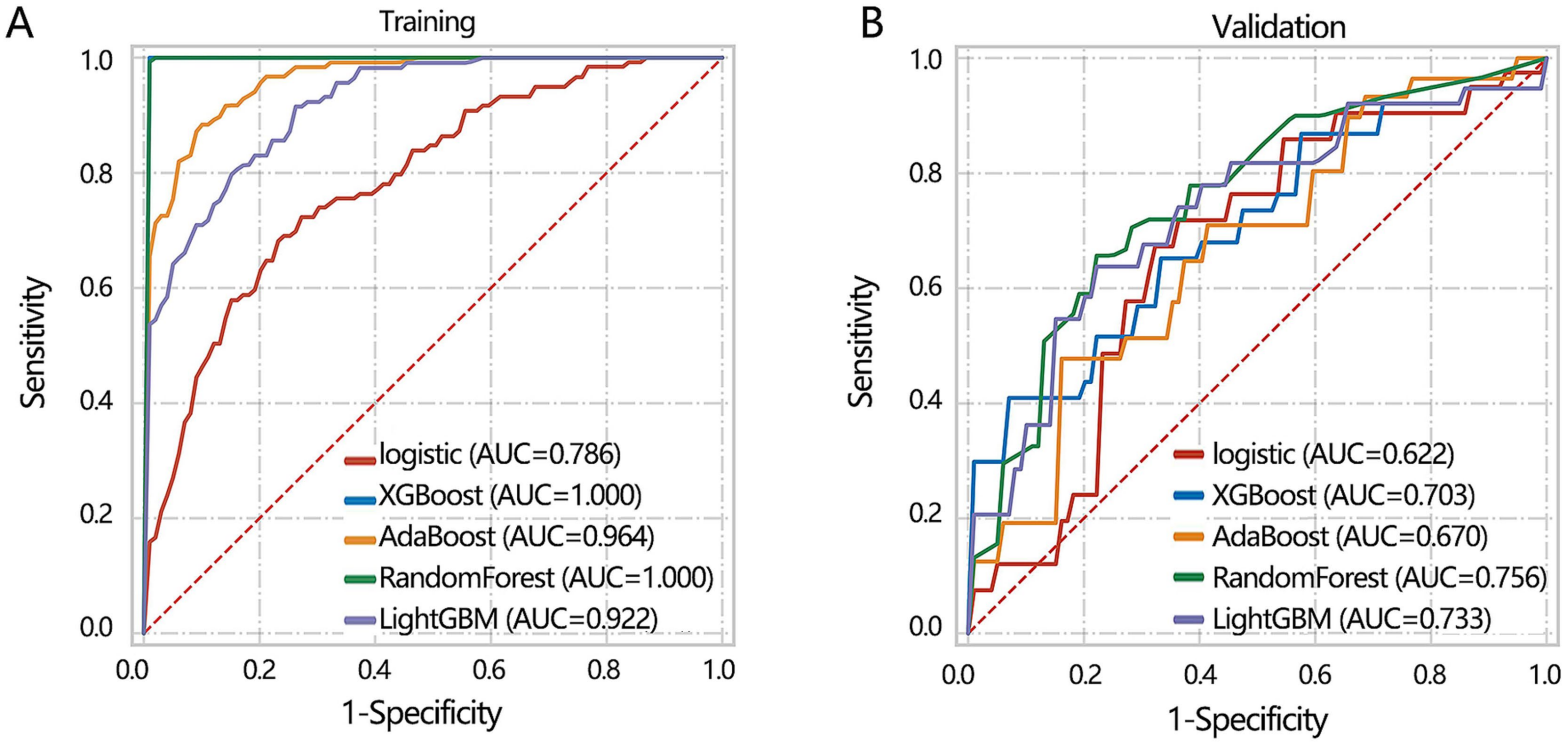
The machine learning model construction and validation based on 3 clinical indicators including age, IL-6, and gender. **(A)** Training set. **(B)** Validation set.

Subsequently, we aimed to construct a clinical feature-based differentiation model. Before model construction, all patients were assigned to training and validation sets. The 3 clinical feature-based differentiation models were first constructed in the training set using 5 deep learning algorithms, and then the differentiation performance of the model was verified in the validation set. The results showed that XGBoost and RandomForest had the most favorable differentiation performance in the training set (Figure 4A). In the validation set, the RandomForest algorithm achieved the highest AUC value (Figure 4B). Integrating the results of the training set and validation set, XGBoost algorithm may show the overfitting and RandomForest algorithm had more stable differentiation performance. Hence, 3 clinical feature-based models by the RandomForest algorithm were regarded as the optimal deep learning model.

### 3.3 Radiomics features-based differentiation model

**Figure 5:**
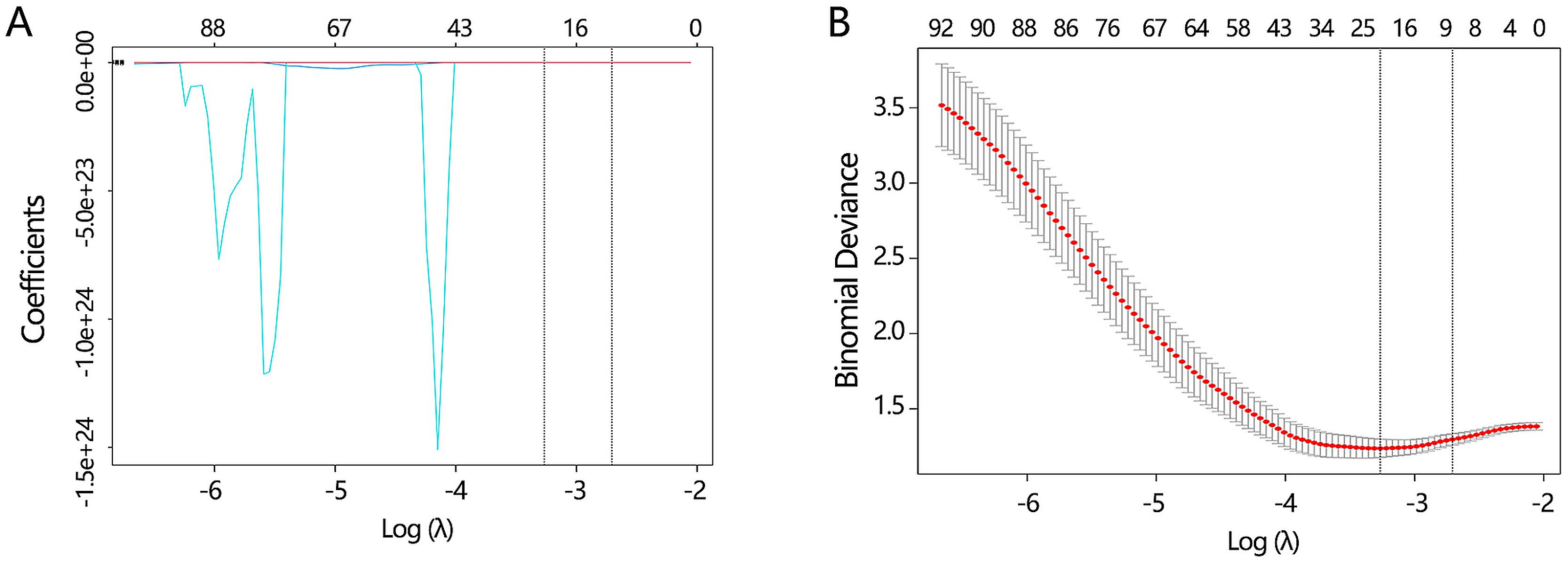
The key radiomics features selection. **(A)** The LASSO analysis was conducted to filter the redundant features among all radiomics features. **(B)** The optimal LASSO model was obtained when the standard error of minimum distance presented the *λ* = 0.067.

Besides the key clinical features, the CT images can also provide some valuable information for disease differentiation. To reveal the potential value of CT images, we explored significant radiomics features contributing to disease differentiation behind the CT images. A total of 837 radiomics features were extracted. To identify the most valuable features among 837 features, we performed the LASSO analysis to remove the redundant features by compressing their coefficients to 0. The optimal LASSO model (Figures 5A,B) was obtained when the standard error of minimum distance presented the *λ* = 0.067, containing 7 non-zero radiomics features. The detailed information on these 7 radiomics features is shown in Table 2.

**Table 2:** The differences of 7 radiomics features between 2 groups.

Features	Image type	Feature class	Feature name	TB (*n* = 99)	NTM (*n* = 76)	*p*
X75	Original	GLSZM	Gray Level Variance	5.617 ± 1.260	5.047 ± 1.300	0.007
X210	wavelet-HLL	GLCM	Correlation	0.121 [0.066, 0.195]	0.200 [0.122, 0.282]	<0.001
X387	wavelet-HLH	FIRSTORDER	Skewness	−0.182 [−0.390, −0.014]	−0.303 [−0.466, −0.124]	0.014
X539	wavelet-HLH	GLSZM	GrayLevelNonUniformityNormalized	0.516 [0.504, 0.541]	0.523 [0.510, 0.565]	0.046
X640	wavelet-HLH	GLSZM	SizeZoneNonUniformityNormalized	0.460 [0.424, 0.561]	0.430 [0.394, 0.505]	0.006
X709	wavelet-HLH	GLSZM	GrayLevelNonUniformityNormalized	0.500 [0.500, 0.500]	0.500 [0.500, 0.500]	0.097
X751	wavelet-LLL	FIRSTORDER	Maximum	745.900 [735.390, 765.304]	755.041 [744.533, 767.961]	0.022

Among 7 features, X709 (GLSZM-Gray Level Non-Uniformity Normalized) showed no difference between the 2 groups (Table 2). The remaining 6 features were further found to correlate with the disease type in univariable logistic regression analysis (Table 3, all *p* < 0.05), and multivariable regression analysis showed that X75 (GLSZM, Gray Level Variance), X210 (GLCM, Correlation), and X751 (FIRSTORDER, Maximum) were independent factors of disease type (all *p* < 0.05). It should be stated that the confidence interval of X210 was significantly abnormal, hence X210 was removed from our analysis. Finally, only X75 and X751 features were entered into our further analyses.

**Table 3:** The association of related radiomics features with disease type.

	Univariable		Multivariable	
Features	OR (95%CI)	*p*	OR (95%CI)	*p*
X75	0.704 [0.542, 0.915]	0.009	0.636 [0.446, 0.886]	0.009
X210	168.781 [6.789,4195.777]	0.002	114.373 [2.273, 7595.81]	0.021
X387	0.357 [0.142, 0.899]	0.029	0.499 [0.180, 1.169]	0.137
X539	11.532 [9.205, 14.447]	0.015	6.157 [0.138, 13.969]	0.113
X640	0.013 [0.001, 0.250]	0.004	0.048 [0.001, 1.670]	0.110
X751	1.019 [1.003, 1.035]	0.020	1.035 [1.014, 1.060]	0.003

The features information can be found in Table 2.

**Figure 6:**
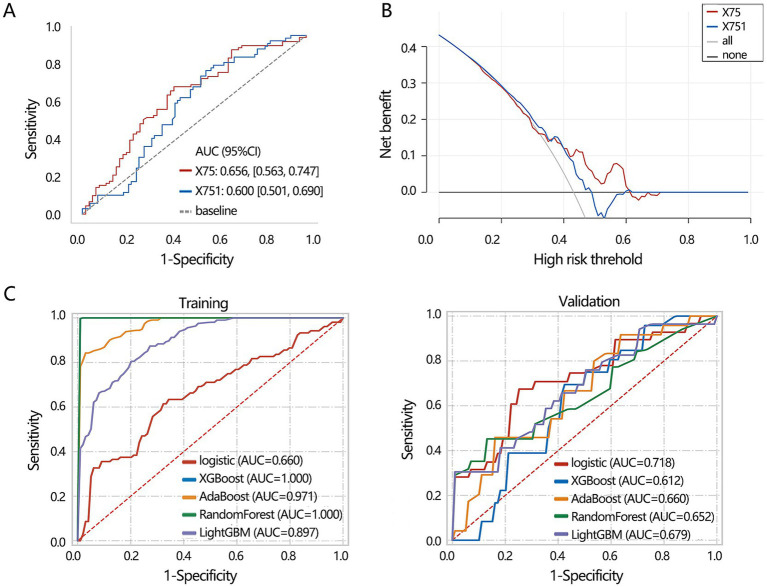
Machine learning model construction based on 2 radiomics features including X75 and X751. **(A)** ROC analysis was conducted to assess the discriminating performance of single radiomics features. **(B)** DCA analysis was used to assess the clinical net benefit of 2 radiomics features for discriminating disease. **(C)** Machine learning model construction and validation based on 2 radiomics features. X75: Gray Level Variance (GLSZM); X751: Maximum (FIRSTORDER).

ROC analysis showed that X75 had a higher AUC value for discriminating the disease than the X751 (Figure 6A, P for Delong test<0.001), with a sensitivity of 0.713 and specificity of 0.591. The obtained clinical net benefit of X75 was also superior to that of X751 (Figure 6B). Based on X75 and X751, a 2 radiomics features-based differentiation model was constructed using 5 deep learning algorithms in our training set, and the differentiation performance of the model was verified in the validation set. In the training set, XGBoost and RandomForest algorithms achieved the highest AUC values, while they achieved the lowest differentiation performance in the validation set (Figure 6C). It followed that the Logistic algorithm achieved a relatively stable performance both in training and validation set. Hence, 2 radiomics features-based model from the Logistic algorithm was regarded as the optimal deep learning model.

### 3.4 Construction of multimodal differentiation model

**Figure 7:**
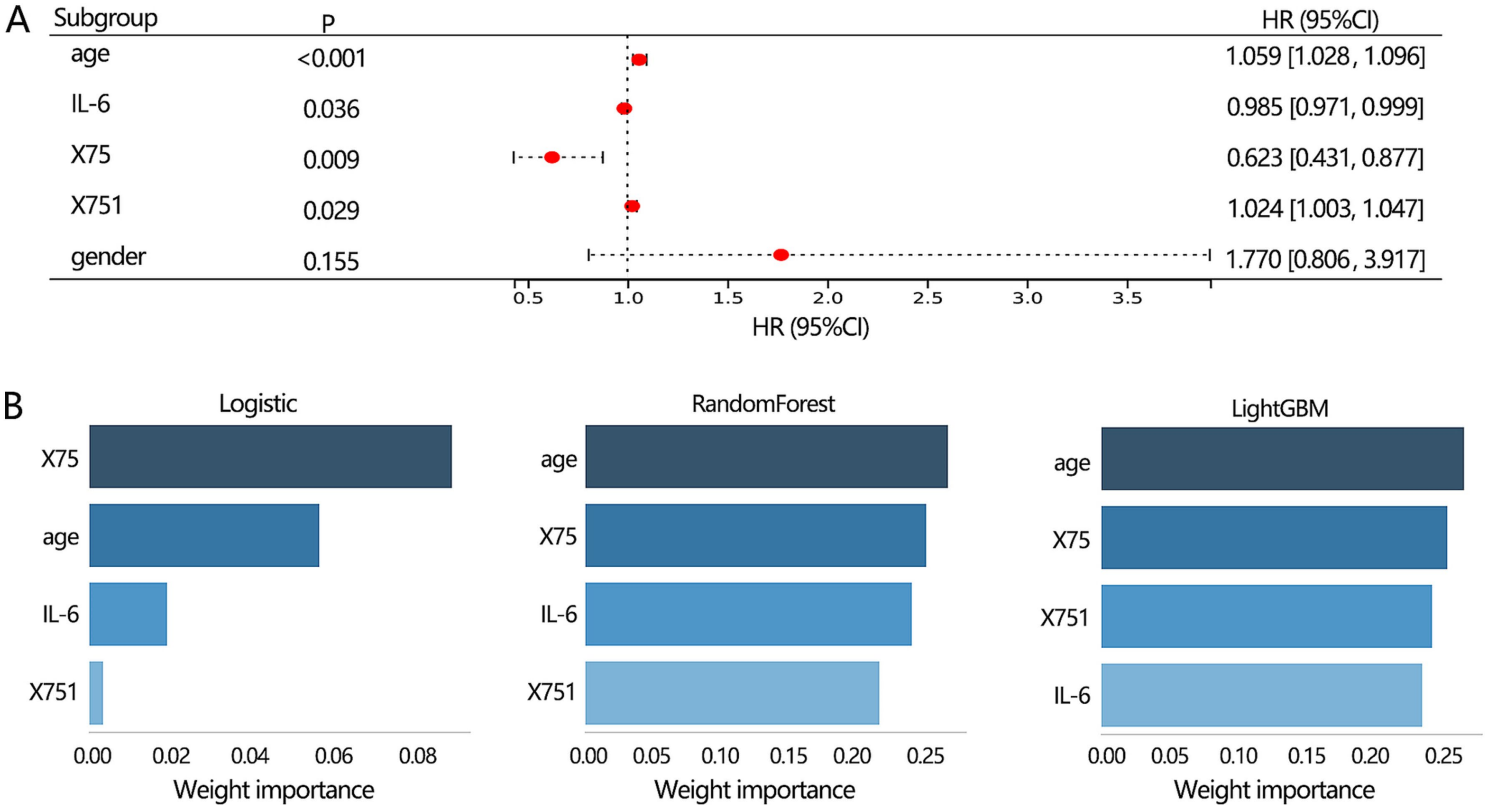
The features selection for constructing the multimodal model construction. **(A)** Multivariable logistic regression analysis on 3 clinical and 2 radiomics features was conducted to identify the proper indicators for constructing the multimodal model. **(B)** The importance ranking of features within the multimodal model.

The above results have demonstrated the importance of 3 clinical features (age, gender, IL-6 level) and 2 radiomics features (X75, X751) in the differentiation of lung disease, respectively. We further combined these 5 features and confirmed their independent role in the disease types. The multivariable regression analysis showed that age, IL-6, X75, and X751 were all independently related to the disease types (Figure 7A). Further, we constructed a multimodal differentiation model based on the 4 features in the training set, followed by verification in the validation set. Considering the model performance in training and validation sets, the LightGBM algorithm achieved a relatively favorable and stable differentiation performance (Table 4). Therefore, the multimodal model constructed by the LightGBM algorithm was regarded as the optimal model.

**Table 4:** Multimodal model construction and validation using 5 machine learning methods.

	Training set					Validation set				
	XGBoost	Logistic	LightGBM	RandomForest	AdaBoost	XGBoost	Logistic	LightGBM	RandomForest	AdaBoost
AUC	1.000	0.808	0.954	1.000	0.999	0.717	0.699	0.804	0.755	0.738
Cutoff	0.741	0.452	0.522	0.575	0.500	0.741	0.452	0.522	0.575	0.500
Accuracy	0.991	0.750	0.895	0.987	0.978	0.690	0.672	0.724	0.655	0.655
Sensitivity	1.000	0.792	0.857	0.990	0.990	0.750	0.811	0.875	0.893	0.844
Specificity	1.000	0.726	0.943	0.992	0.985	0.700	0.643	0.698	0.600	0.577
PPV	1.000	0.716	0.926	1.000	0.979	0.762	0.608	0.673	0.833	0.708
NPV	0.984	0.785	0.872	0.977	0.977	0.675	0.752	0.738	0.609	0.604
Kappa	0.982	0.498	0.786	0.973	0.955	0.374	0.351	0.409	0.296	0.307

NPV, Negative predictive value; PPV, positive predictive value.

Our results showed that the best 3 clinical features-based models, 2 radiomics features-based models, and the multimodal model were from RandomForest algorithm, Logistic algorithm, and LightGBM algorithm, respectively. Therefore, we next used the 3 algorithms to rank the importance of 4 features within the multimodal model. The results showed (Figure 7B) that age and X75 were the top 2 features among the 3 algorithms.

### 3.5 The performance comparisons of different models for differentiating NTM from MTB

**Figure 8:**
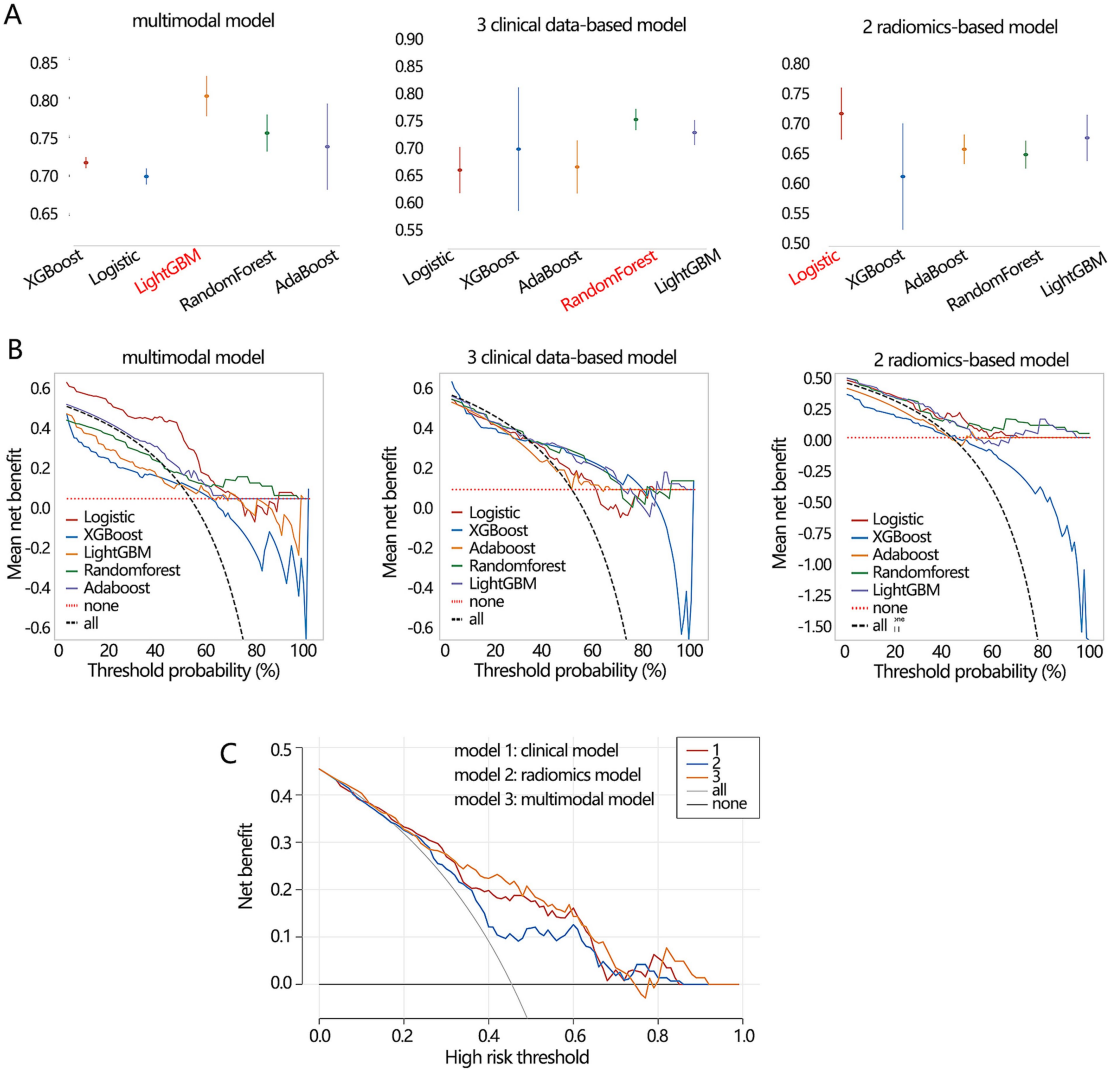
The performance comparisons on 3 types of differentiation model. **(A)** The AUC value and **(B)** clinical net benefit comparisons of 3 models constructed by 5 algorithms in the validation set. The red font implied the optimal model. **(C)** The obtained clinical net benefit of 3 differentiation models based on Logistic regression analysis among whole populations.

Finally, we compared the differentiation performance of 3 optimal models constructed by corresponding algorithms only in the validation set due to the stability consideration. The optimal multimodal model from the LightGBM algorithm (Figure 8A) had the highest AUC value (0.804) compared with the optimal clinical model (0.756) and radiomic model (0.718). In addition, the optimal multimodal model (Table 5) had the highest accuracy (0.724), sensitivity (0.875), and NPV (0.738). However, the multimodal model had the lowest specificity (0.693). The PPV values of the optimal multimodal model was in between the clinical model and the radiomics model. The clinical net benefit of 3 models seemed to be similar (Figure 8B). To better visualize the net benefit, we next performed the DCA analysis only based on the Logistic regression (Figure 8C) among the whole population, finding that the multimodal model had better clinical net benefit.

**Table 5:** The comparisons among different differentiation model.

	3 Clinical data model (RandomForest)		2 Radiomics model (Logistic)		Multimodal model (LightGBM)	
	Training	Validation	Training	Validation	Training	Validation
AUC	1.000	0.756	0.660	0.718	0.954	0.804
Cutoff	0.500	0.500	0.442	0.442	0.522	0.522
Accuracy	0.981	0.712	0.652	0.677	0.895	0.724
Sensitivity	1.000	0.690	0.615	0.708	0.857	0.875
Specificity	0.993	0.767	0.693	0.733	0.943	0.698
PPV	1.000	0.728	0.589	0.633	0.926	0.673
NPV	0.966	0.707	0.699	0.719	0.872	0.738
kappa	0.961	0.425	0.288	0.353	0.786	0.409

NPV, negative predictive value; PPV, positive predictive value.

### 3.6 Performance verification and comparison using the external testing dataset

We further verified the prediction performance among 3 clinical data based-model, 2 radiomics based-model, and multimodal model for differentiating NTM from MTB using a new external testing dataset. There were 36 male and 23 female patients in the new external testing dataset. The median age, IL-6, X75, and X751 were 61 [52, 67] years old, 10.9 [6.4, 33.2] (pg/mL), 4.77 [3.68, 5.42], and 766 [751, 771], respectively. The comparison results showed that the multimodal model had the highest AUC, accuracy, sensitivity, and NPV, but had the lowest specificity (Table 6). These results were similar to those from the validation set, which suggested the stability of our findings.

**Table 6:** The performance comparison of different models for differentiating NTM and MTB in the external testing dataset.

	3 Clinical data model (RandomForest)	2 Radiomics model (Logistic)	Multimodal model (LightGBM)
AUC	0.657	0.766	0.858
cutoff	0.550	0.310	0.204
Accuracy	0.583	0.744	0.745
Sensitivity	0.200	0.833	0.900
Specificity	0.857	0.710	0.667
PPV	0.500	0.526	0.581
NPV	0.600	0.917	0.929

AUC, area under curve; NPV, negative predictive value; PPV, positive predictive value.

**Figure 9:**
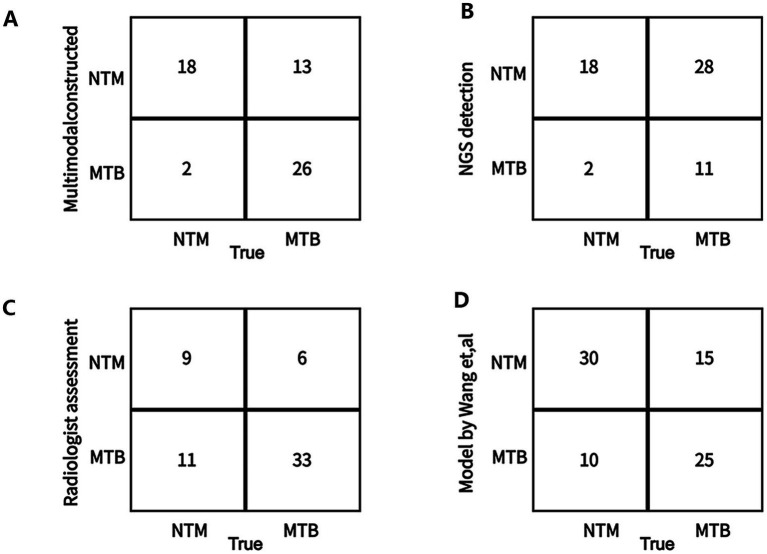
Confusion matrix on the test between the constructed multimodal model and other approaches for differentiating NTM from MTB in the external testing dataset. **(A)** Our multimodal model. **(B)** Only Next-generation sequencing (NGS) detection in this study. **(C)** Only radiologist assessment in this study. **(D)** A deep learning model (published by Want et al.) using CT images.

We also compared the differentiation performance of our multimodal model with the only NGS detection method, only radiologist assessment, and existing machine learning model constructed by Wang et al. (33) for differentiating NTM from MTB. The results (Table 7; Figure 9) showed that our multimodal model had the highest AUC, accuracy, and sensitivity. The NGS detection also had favorable sensitivity. Radiologist assessment had the highest specificity and favorable accuracy. The published model by Wang et al. using CT images only had the highest precision for identifying NTM (yes vs. no). Our multimodal model improved accuracy than NGS, radiologist, and existing machine learning model, with an increased accuracy of 26, 4, and 6%, respectively. It also significantly improved sensitivity than radiologist and existing machine learning model, with an increased sensitivity of 4.5 and 15%, respectively, but was similar with that of NGS detection. These results highlighted the superiority of our multimodal model for differentiating NTM from MTB compared with existing approaches or existing machine learning model. Our model significantly improved the differentiation performance and accuracy, and provided favorable sensitivity at the same time.

**Table 7:** The performance comparison between the multimodal model and other approaches for differentiating NTM from MTB in the external testing dataset.

	Our model	Only NGS detection	Only radiologist	Published model by Wang et al.
Details	Multimodal constructed	DNA sequencing	5-year experience	Only CT images
AUC	0.86	0.59	0.65	0.78
Accuracy	0.75	0.49	0.71	0.69
Sensitivity	0.90	0.90	0.45	0.75
Specificity	0.67	0.28	0.85	0.63
MTB-Precision	0.93	0.85	0.75	0.71
NTM-Precision	0.58	0.39	0.60	0.67

AUC, area under curve; NGS, Next-generation sequencing.

## 4 Discussion

In this study, we initially established an optimal 3 clinical features-based differentiation model. We also constructed an optimal 2 radiomics features-based differentiation model. The differentiation performance of the clinical model was superior to the radiomics model. Finally, we developed an optimal multimodal differentiation model containing clinical and radiomics data. After analysis, our multimodal differentiation model showed more favorable differentiation performance compared with the single clinical or radiomics model. Our study suggested the necessity of the combination of clinical data and radiomics data in disease differentiation.

In our multimodal differentiation model, 4 key features were contained including age, IL-6, and 2 radiomics features. Spatial epidemiologic analysis showed that a higher risk for NTM infection was associated with older age, rurality, and more flooding (34). The previous study showed that females were 1.4 times more likely to infect NTM than males, clustering persons with age ≥ 65 years (35). We found that the NTM patients had a larger age compared with MTB patients and the median age of NTM patients was 66 years old, which was consistent with the previous findings. NTM infections have become a neglected and emerging problem in geriatric patients, and the older adult population is more susceptible to NTM and experiences increased morbidities (36). Our study and other findings (33, 37, 38) all suggested that NTM was more common in older adult people, which may be because NTM was a type of opportunistic pathogen, and patients infected with NTM often developed symptoms due to their aging and weakened immune system. However, we found no difference in gender distribution between the NTM and MTB groups, although our results were consistent with the previous study (33). It follows that patient characteristics alone are insufficient in differentiating between NTM and MTB.

It should be stated that NTM-infected old mice had significant dysrhythmia, cardiac hypertrophy, cardiac fibrosis, and elevated CD45^+^ leukocyte levels and expression of inflammatory genes in heart tissue (39). It follows that NTM infections may contribute to cardiac dysfunction in the older adult population, which is a cause for concern. Besides the involvement of elevated age in the NTM infection, this study also found a decreased IL-6 level in the NTM group. The previous study (40) also reported that the production of IL-6 in the NTM infection group occurred to a significantly lesser extent, and p38 and extracellular regulated protein kinases (ERK1/2) played essential roles in the production of IL-6 during NTM infection. The impaired induction of p38 and ERK1/2 expression in response to NTM may contribute to host susceptibility to NTM lung disease. In addition, the level of IL-6 in macrophages infected with NTM can also be regulated by XLOC_002383/miR-146a-5p/TRAF6 axis (TRAF6, TNF receptor associated factor 6) (41). IL-6 has been regarded as a biomarker for discriminating NTM and other lung disease.

In addition, we also obtained 2 important radiomics features for discriminating lung disease. Especially, the importance of GLSZM [Gray Level Variance] (X75) was highlighted. Currently, there is no study reporting the role of GLSZM [Gray Level Variance] in the discrimination of NTM lung disease. The texture features represented by GLSZM mainly reflected the variability (heterogeneity of image texture) in the measurement area, and lower values indicated that the regions in the image are more homogeneous (42). Gray Level Variance is a regional-scale heterogeneity indices derived from GLSZM. In this study, NTM group had higher Gray Level Variance value, suggesting the higher heterogeneity in NTM than in MTB. In addition, Gray Level Variance value had favorable performance for differentiating NTM from NTM. The results suggested that distinctive textural features between NTM and MTB could be better captured by regional-scale lesion heterogeneity. Imaged heterogeneity may be due to the regional differences in cellularity, proliferation, hypoxia, angiogenesis, and necrosis (43). The previous study indicated that decreased lung tissue oxygenation may contribute to the development of NTM disease (44), and the level of hypoxia in NTM lesions in mice was more severe than that observed in the setting of tuberculosis. It followed that the hypoxia level presented regional differences between NTM and TB. In addition, NTM and TB also showed the difference in hematological profiles. A pilot study found that TB patients had higher basophils and platelets levels, but lower eosinophils level than NTM patients (45). Moreover, MTB-infected cells had lower level of phagosome-lysosome fusion and apoptosis than NTM-infected cells (46). We speculated that the final selection of GLSZM [Gray Level Variance] as key features may be related to the heterogeneity in hypoxia, blood, etc. between NTM and MTB. NTM infections are common but are often cofounded with TB because of the similarity of symptoms, therefore, it is necessary to find more heterogeneity between them.

The previous study (13) also established a machine learning-based differentiation of NTM and MTB using CT images, finding that feature extracting from bronchiectasis was relatively more informative than that from a cavity or the combination (bronchiectasis+ cavity). This study highlighted the effectiveness difference of different regions (cavities, bronchiectasis, and their combination). In addition, chest X-rays from suspects of mycobacterial lung disease were also used to distinguish between TB or NTM patients by artificial intelligence, finding that deep neural networks had a better performance than pulmonologists on classifying patients (47). It follows that the deep learning model may achieve favorable differentiation performance, and a more effective model needs to be investigated.

Finally, several limitations should be stated. In this study, the construction of the multimodal model depended on high-quality CT imaging, and the sample size was not sufficiently large, which may influence the generalizability and application of the models. The DCA was used to evaluate the clinical net benefit of 3 models, but it was only based on Logistic regression and we failed to use more methods to validate the clinical net benefit difference due to different algorithms. In addition, we just verified the model performance by an external testing dataset from our hospital and the external dataset lacks diversity, which may have a selection bias and limit the generalizability of our findings. Another limitation is the absence of comparison with existing clinical methods or diagnostic tools. Without this comparison, it is difficult to assess the added value of the proposed approach. The model validation and comparison are the other key research topic in the future.

## 5 Conclusion

This study developed a multimodal learning model to classify NTM from MTB, with greater accuracy, sensitivity, and negative predictive value than the single clinical or radiomics based models. Our multimodal model improved accuracy than NGS, radiologist, and existing machine learning model. It also significantly improved sensitivity than radiologist and existing machine learning model. These results highlighted the superiority of our multimodal model for differentiating NTM from MTB compared with existing approaches. Our study can promote the application of new technologies, thereby advancing research progress in this fields. This is the first study to consider multidimensions and use multimodal model to distinguish diseases, which provides a new perspective and strategy for differentiating NTM from MTB, and help doctors to choose appropriate treatment plans.

## Data Availability

The raw data supporting the conclusions of this article will be made available by the authors, without undue reservation.
